# Age-related changes in the *BACH2* and *PRDM1* genes in lymphocytes from healthy donors and chronic lymphocytic leukemia patients

**DOI:** 10.1186/s12885-019-5276-2

**Published:** 2019-01-17

**Authors:** Vu Luan Dang Chi, Soizic Garaud, Pushpamali De Silva, Vincent Thibaud, Basile Stamatopoulos, Mimoune Berehad, Chunyan Gu-Trantien, Mohammad Krayem, Hugues Duvillier, Jean-Nicolas Lodewyckx, Karen Willard-Gallo, Catherine Sibille, Dominique Bron

**Affiliations:** 1 Clinical and Experimental Hematology, Institut Jules Bordet, Université Libre de Bruxelles, Rue Heger Bordet 1, 1000 Brussels, Belgium; 2 Molecular Immunology Unit, Institut Jules Bordet, Université Libre de Bruxelles, Brussels, Belgium; 3 Laboratory of Clinical Cell Therapy, Institut Jules Bordet, Université Libre de Bruxelles, Brussels, Belgium; 4 Flow Cytometry Core Facility, Institut Jules Bordet, Université Libre de Bruxelles, Brussels, Belgium; 5 Institut of Medical Immunology, Université Libre de Bruxelles, Brussels, Belgium; 6 Department of Pathology, Institut Jules Bordet, Université Libre de Bruxelles, Brussels, Belgium; 7 Laboratory of Oncology and Experimental Surgery, Institute Jules Bordet, Université Libre de Bruxelles, Brussels, Belgium

**Keywords:** BACH2, PRDM1, Immunosenescence, Chronic lymphocytic leukemia, Lymphocytes, Apoptosis

## Abstract

**Background:**

Age-related genetic changes in lymphocyte subsets are not currently well documented. BACH2 is a transcription factor that plays an important role in immune-mediated homeostasis by tightly regulating PRDM1 expression in both B-cells and T-cells. *BACH2* gene expression is highly sensitive to DNA damage in aged mice. This concept led us to investigate the variation in BACH2 and also PRDM1 expression in major lymphocyte subsets with age.

**Methods:**

Lymphocyte subsets from 60 healthy donors, aged from 20 to 90 years, and 41 untreated chronic lymphocytic leukemia patients were studied. *BACH2* and *PRDM1* gene expression was analyzed by real-time quantitative PCR. *BACH2* gene expression was correlated with its protein expression. Lymphocyte apoptosis was evaluated after intracellular oxidative stress-inducing etoposide treatment of T and B cells.

**Results:**

Our analysis shows *BACH2* mRNA downregulation with age in healthy donor CD4+, CD8+ T-cells and CD19+ B-cells. Decreased BACH2 expression was also correlated with an age-related reduction in CD8 + CD28+ T-cells. We found a strong correlation between age-related *BACH2* downregulation and decreased CD4+ T-cell and CD19+ B-cell apoptosis. *PRDM1,* as expected, was significantly upregulated in CD4+ T-cells, CD8+ T-cells and CD19+ B-cells, and inversely correlated with *BACH2*. A comparison of untreated chronic lymphocytic leukemia patients with age-matched healthy donors reveals that *BACH2* mRNA expression was further reduced in CD4+ T-cells, CD8+ T-cells and leukemic-B cells. *PRDM1* gene expression was consequently significantly upregulated in CD4+ and CD8+ T-cells in chronic lymphocytic leukemia patients but not in their leukemic B-cells.

**Conclusion:**

Overall, our data suggest that *BACH2* and *PRDM1* genes are significantly correlated with age in human immune cells and may be involved in immunosenescence.

**Electronic supplementary material:**

The online version of this article (10.1186/s12885-019-5276-2) contains supplementary material, which is available to authorized users.

## Background

Aging is a complex, multistep process with a highly variable course of progression that leads to a higher predisposition to infections, cancer and autoimmune diseases in the elderly. Bhatia-Dey N., et al. proposed that aging results from the sequential passage through three distinct phases: (a) molecular damage leading to (b) the cessation of proliferation, which causes cellular senescence, followed by (c) body-wide aging of the organism [1]. However, cellular senescence and apoptosis are two types of cellular responses to DNA and cellular damage that are altered in both cancer (BACH2 repression in CD4+ T cells modulates their resistance to apoptosis demonstrating it functions as a tumor suppressor gene, in preparation) and aging [2]. The permanence of the senescence growth arrest enforces the idea that the senescence response evolved at least in part to suppress the development of cancer [3].

Advancing age is associated with functional alterations in multiple organs, including the immune system [4–7]. This age-linked remodeling of immunity termed immunosenescence is characterized by a gradual decline in innate and adaptive immune responses which leads to an imbalance in immune homeostasis. As a consequent of immunosenescent related lymphocyte deficiencies including the shrinking of naïve T-cell and B-cell compartments, reduced T-cell and B-cell receptor diversity and decreased T-cell receptor sensitivity to stimuli, immunosurveillance decreases [4–8]. An age-related proinflammatory state is highlighted by excessive production of inflammatory cytokines such as IL-6, IL-4, IFNγ and TNF and auto-antibodies [7, 9–11].

Aging also favors the accumulation of genetic and epigenetic changes that are important contributing factors to the development of cancer [12]. Accumulating evidence shows that senescent cells can have a deleterious effect on the tissue microenvironment. The hallmark of these effects is the acquisition of a senescence-associated secretory phenotype that turns senescent fibroblasts into proinflammatory cells with the ability to promote tumor progression [13]. Concomitantly, many other factors favor cancer development in older patients, such as decreased apoptosis, micro-environmental damages, chromosomal instability and exposure to carcinogens [14]. However, a full understanding of the aging process is far from complete with many open questions currently under investigation [15].

The transcription factor BACH2 (BTB and CNC homology 1, basic leucine zipper transcription factor 2) has been suggested as a marker of DNA damage and aging [16]. This was demonstrated by the experiments done in mice which showed, *BACH2* gene expression is highly sensitive to transcription-blocking in DNA lesions caused by UV irradiation in dermal fibroblasts from aged mice [16]. BACH2 has been shown to be involved in B-cell and memory CD4+ T-cell differentiation and inhibit effector cell functions by limiting antigen-receptor-stimulation-induced gene expression and restricting premature expression of the transcriptional regulator PRDM1 (PR domain zinc finger protein 1) [17]. PRDM1 is necessary for terminal differentiation of antibody-secreting plasma cells, while in T-cells, it has been shown to regulate homeostasis of effector and memory CD4+ T-cells [18]. Moreover, the BACH2 protein is retained in the cytoplasm until oxidative stress (oxidative stress damages cells and activates defensive responses) induces its nuclear translocation and accumulation, which ultimately provokes apoptosis [19–22].

Chronic lymphocytic leukemia (CLL) is a B lymphocyte malignancy occurring in elderly people (median age at diagnosis of 72 years and median age at death of 79 years) [23] where the tumor cells depend on extracellular stimuli for their survival and behavior [24]. The major consequence of antigen engagement in CLL appears to be anergy, which is observed in all CLL samples but is variable [25]. This could be due to a compromise of the pre-B cell receptor contributing to B-cell repertoire alterations in old age as it has been shown in aged mice [26], which needs further evaluations in CLL patients. CLL-specific clinical data are very limited for predicting therapy-related morbidity, treatment compliance and non-treatment-related mortality. Biomarkers of frailty specifically in CLL are also lacking. A CLL consensus initiative is in progress to help guide CLL-specific fitness scoring [27].

In this study, we prospectively examined BACH2 expression and correlated this with apoptosis in the major lymphocyte subsets from healthy donors (HDs) and CLL patients to evaluate its potential as a predictive marker of aging.

## Methods

### Human samples

All blood samples were collected after written informed consent, in accordance with Institutional Guidelines and the Declaration of Helsinki. The study was approved by the Jules Bordet Institute’s Ethical Committee (CE2324).

Peripheral blood samples were obtained from 60 healthy volunteers (58% male) and 41 untreated CLL patients (60% male). HDs, between the ages of 20 to 90 years, were selected based on clinical records and laboratory examinations. “Healthy” was defined as the absence of acute illness, neoplastic or autoimmune diseases and any medication that could interfere with immune function. The diagnosis of CLL was based on the absolute lymphocyte count in the peripheral blood, lymphocyte immunophenotyping, bone marrow smear and trephine biopsy which was according to the World Health Organization classification of lymphoid neoplasms [28]. Table 1 shows the age distribution of both populations.

**Table 1:** Age distribution of healthy donors and chronic lymphocytic leukemia patients

Age	20–29	30–39	40–49	50–59	60–69	70–79	80-	Total
Healthy Donors								
Number	9	10	8	11	14	5	3	60
Chronic Lymphocytic Leukemia Patients								
Number	0	0	0	7	20	8	6	41

### Lymphocyte subpopulations purification

Peripheral blood mononuclear cells (PBMCs), isolated from HD blood or CLL patients using Lymphoprep™ (STEMCELL Technologies) density gradient centrifugation, were immediately used for lymphocyte subset selection. Lymphocyte subsets were isolated from PBMCs by magnetic labeling and separation using the MACS CD56+ NK cells, CD19+ B-cell, CD4+ and CD8+ T-cell, CD4+ naïve T-cell, CD4+ effector memory T-cell and B-CLL cell isolation kits, respectively, according to the manufacturer’s instructions (Miltenyi Biotech). The purity of the subpopulations was evaluated by flow cytometry, as described below.

### Real-time quantitative PCR (RT-qPCR)

Total RNA was extracted from purified lymphocyte subsets by using TRIzol Reagent (Invitrogen). The quantity of mRNA was assessed by NanoDrop 1000 spectrophotometer. Isolated RNA was reverse transcribed into cDNA using High Capacity RNA-to-cDNA (Applied Biosystems) following standard procedures.

RT-qPCR reactions were performed using Ssoadvanced™ SYBR® Green Supermix with ROX (Bio-Rad) on an ABI 7900HT Prism sequence detector (Applied Biosystems). The relative mRNA expression levels were normalized by the mean of two reference genes, CAS3 and EF1α, and fold changes were calculated using the 2^−ΔCt^ method. All RT-qPCR reactions were carried out in duplicates. Details of PCR primers used are described in Additional file 1: Table S1.

### Flow cytometry

For surface staining, PBMCs (1 × 10^6^ cells in 400 μl PBS containing 1% serum and 0.1% NaN_3_) were incubated with 1 μg antibody in the dark at 4 °C for 30 min and then washed twice. Antibodies used were as follows: CD45, CD19, CD20, CD56, CD16, CD3, CD4, CD45RA, CD45RO, CD197, CD161, CD25 from Miltenyi and CD5, CD8, CD27 from Beckman Coulter (antibody details shows in Additional file 2: Table S2). Fluorescently labeled cells were acquired on a GALLIOS 10/3 cytometer and analyzed using Kaluza® 1.3 Flow Analysis Software (Beckman Coulter).

### Western blot analysis

Total proteins were extracted using Triton X-100 buffer containing a 0.2 mM protease inhibitor cocktail (Thermo Scientific™). Proteins were fractionated by 10% sodium dodecyl sulfate-polyacrylamide gel electrophoresis and then transferred electrophoretically to a PVDF membrane (Amersham) by wet blotting. The membrane was incubated in blocking buffer (Amersham), then with a rabbit anti-BACH2 monoclonal antibody [1:1000, Cell Signaling (D3T3G)]. Endogenous control was anti-beta-actin antibody [(1:1000, Cell Signaling (8H10D10)]. Labeling was detected using enhanced chemiluminescence reagents (Amersham) and analyzed on a ChemiDocTM XRS reader (Bio-Rad).

### Apoptosis assay

PBMCs were seeded in 12 well plates at the density of 1 × 10^6^ cells per ml per well. After incubation for 24 h at 37 °C with 50 μM of etoposide (Sigma™) to induce oxidative stress, cells were harvested and washed twice with PBS-EDTA. Apoptotic cells were identified with the annexin-V-FITC apoptosis detection kit from BD Pharmingen kit (556547) following the manufacturer’s instructions. PBMCs were immunostained for CD45, CD19 (eBioscience) or CD3, and CD4 (Miltenyi) prior to annexin-V-FITC/IP staining. The apoptotic cells were analyzed by flow cytometry, as described above.

### Statistical analysis

All statistical analyses were performed with GraphPad Prism 5.0 software. Statistical significance was calculated using an unpaired Mann-Whitney test. Spearman’s correlation (r) was used to measure the correlation between sets of data. Statistical significance was defined as *p* <  0.05.

## Results

### The impact of age on HD lymphocyte subset distribution

We analyzed phenotypic changes in the major lymphocyte subsets by age groups. The age-related quantitative analysis of lymphocyte subpopulations in our study (Table 2) confirms previous reports (Utsuyama, Kikuchi et al. 2009). There were non-significant age-related changes in the absolute cell counts for CD3+, CD4+ and CD19+ lymphocyte subpopulations. We found three important observations; [1] age-related increase in CD4/CD8 ratio, reduction of the number of naïve CD4+ and increase in effector memory CD4+ T-cells with age, and [2] a significant decline in the number of cytotoxic T-cells (both CD8+ and CD8 + CD28+) (Additional file 3: Table S3).

**Table 2:** Regression analysis of healthy donor’s BACH2 mRNA expression versus the number of CD8+ T-cells or CD8 + CD28+ T-cells

Number of	n	Regression curve	R2 ^a^	*p*-value
CD8+ T-cells	60	+ 26.524x + 281.88	0.344	0.0005
CD8 + CD28+ T-cells	60	+ 36.059x + 76.078	0.4275	< 0.0001

^a^ R2: Correlation coefficient

x: age

### The impact of age on *BACH2* and *PRDM1* gene expression in HD major lymphocyte subsets

**Fig. 1:**
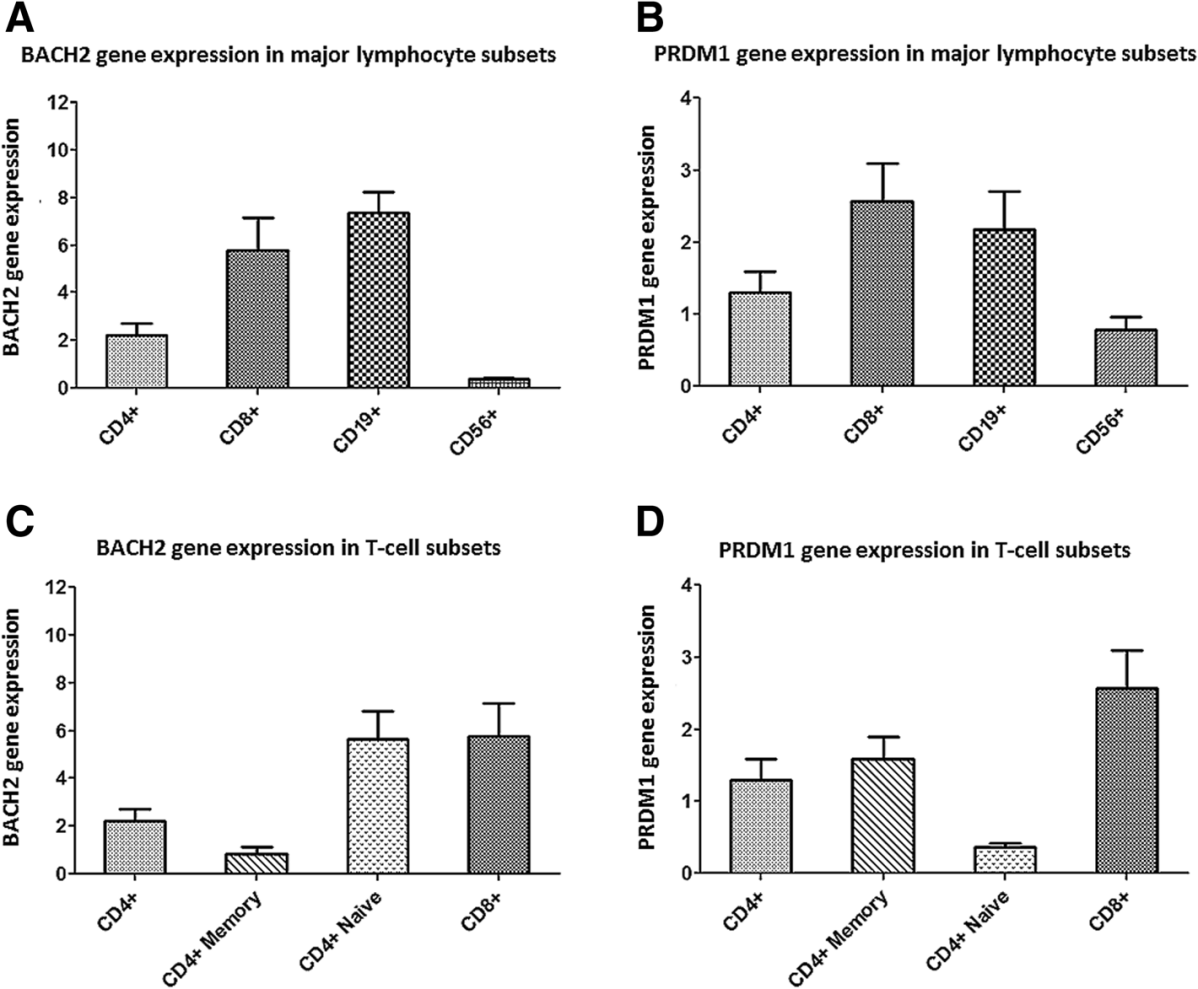
BACH2 and PRDM1 gene expression in the major lymphocyte subsets. Expression level determined by RT-qPCR for the BACH2 (**a** and **c**) and PRDM1 (**b** and **d**) genes in healthy donors’ major lymphocyte subsets. RT-qPCR data are relative to BACH2 and PRDM1 expression (2^-ΔCt^) done in duplicates. Data are expressed as mean ± SD. *n* = 60 for CD4+, CD8+ and CD19+ subpopulations. *n* = 35 for the CD4+ naïve, CD4+ memory and CD56+ subpopulations

We examined *BACH2* and *PRDM1* gene expression changes by age groups in HD major lymphocyte subsets. RT-qPCR showed that *BACH2* mRNA expression was very low in CD56+ NK-cells, whereas it was highly expressed in CD19+ B-cells and CD4+ and CD8+ T-cells (Fig. 1a). BACH2 mRNA expression in total CD4+ T-cells was lower than in CD8+ and CD19+ lymphocytes. Among T-cell subpopulations, naïve CD4+ and CD8+ T-cells showed the highest level of BACH2 expression while CD4+ memory T-cells showed a much reduced level of expression (Fig. 1c). The level of *PRDM1* mRNA expression in CD8+ and CD19+ lymphocytes were much higher than in CD4+ and CD56+ lymphocytes (Fig. 1b), but contrary to BACH2, *PRDM1* mRNA showed a higher expression in CD4+ memory T-cells than in CD4+ naïve T-cells (Fig. 1d).

**Fig. 2:**
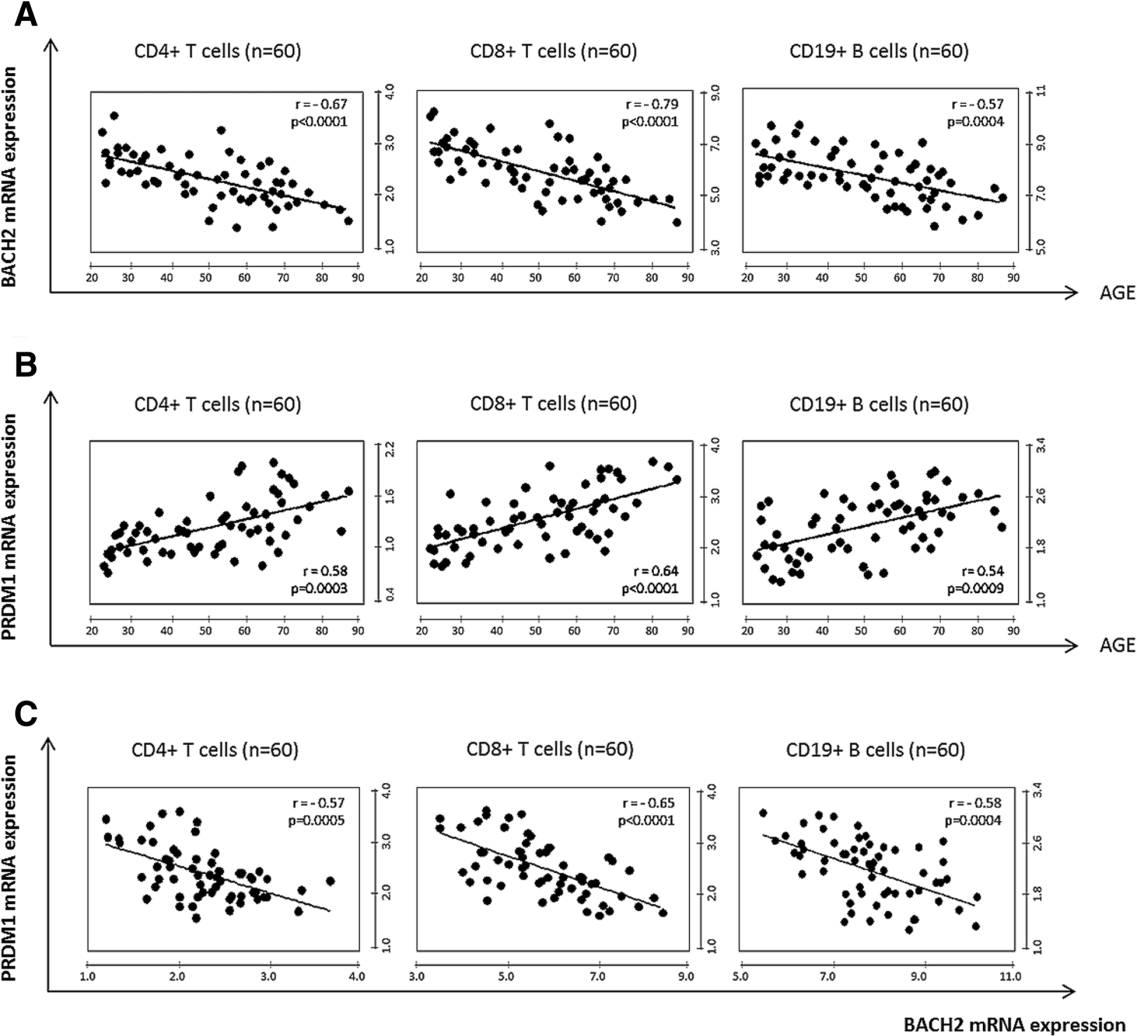
Correlation of BACH2 and PRDM1 with age in healthy donors. **a** BACH2 mRNA expression decreased with age in CD4+, CD8+ and CD19+ (*r* = − 0.67, *r* = − 0.79, *r* = − 0.58, respectively) (**b**) PRDM1 mRNA expression upregulation with age in CD4+, CD8+ and CD19+ (*r* = 0.58, *r* = − 0.64, *r* = − 0.54, respectively). **c** The inverse correlation between BACH2 and PRDM1 mRNA expression in CD4+, CD8+ and CD19+ (*r* = − 0.57, *r* = − 0.65, r = − 0.58, respectively). Significant *p* values (< 0.05) and Spearman r values are shown

*BACH2* and *PRDM1* gene expression were strongly correlated with age in HD major lymphocyte subsets (Fig. 2). BACH2 mRNA expression declined with age, with statistically significant differences observed in all lymphocyte subsets: CD4+, CD4+ naïve, CD4+ memory, CD8+ T-cells and CD19+ B-cells (*p* <  0.0001, < 0.0001, 0.0118, < 0.0001 and 0.0004 respectively) (Fig. 2a and Additional file 4: Table S4). In contrast, *PRDM1* mRNA expression significantly increased with age in the same subpopulations (Fig. 2b and Additional file 5: Table S5). As expected, BACH2 expression was inversely correlated with PRDM1 in CD4+ T-cells, CD8+ T-cells and CD19+ B-cells (*r* = 0.57; 0.65 and 0.58 respectively) (Fig. 2c).

We also noted a strong correlation between *BACH2* mRNA expression with the number and activity of CD8+ T-cells. Decreases in BACH2 expression were proportional to the reduction of CD8+ T-cell and CD8 + CD28+ T-cell counts (*p* = 0.0005 and < 0.0001) (Table 2).

### Age-related BACH2 downregulation and its correlation with the expression of effector memory-related genes in CD4+ T-cells

It has been shown that BACH2 and PRDM1, IL-4, IFNγ are involved in the development and differentiation of lymphocytes, especially CD4+ T-cells. BACH2 plays important roles in immune homeostasis. BACH2 maintains T-cells in the naive state by suppressing effector memory-related genes. T-cells from BACH2 knock-out mice highly expressed Th2-related genes, such as IL-4 and BLIMP-1 (PRDM1) but not IFNγ that highly is expressed in Th1 [29].

Therefore, we examined the latter gene expressions in the CD4+ T-cell subset from our HD cohort. With increasing age, *BACH2* gene expression had decreased while *PRDM1* and *IL-4* genes showed increased expression in CD4+ T-cells. *IFNγ* gene expression showed non-significant change (Additional file 6: Figure S1). Moreover, our analysis showed that age-related BACH2 downregulation inversely correlated with the expression of *PRDM1* and *IL-4* but not *IFNγ* (Table 3).

**Table 3:** Regression analysis of effector memory-related genes: PRDM1, IL-4 and IFNγ versus BACH2 in CD4+ T-cells from healthy donors

mRNA expression in CD4+ T cells	n	Regression curve	R2 ^a^	*p*-value
PRDM1 mRNA expression	60	−0.305x + 1.983	0.3249	0.0005
IL-4 mRNA expression	46	−0.886x + 4.577	0.3713	< 0.0001
IFNγ mRNA expression	46	+ 0.145x + 1.850	0.0352	0.2116

^a^ R2: Correlation coefficient

x: BACH2 mRNA expression in CD4+ T-cells

### Age-related BACH2 downregulation and its correlation with decreased apoptotic cells

Oxidative stress damage activates cellular defensive mechanisms. BACH2 plays a role in oxidative stress-mediated apoptosis, and oxidative stress activates its nuclear accumulation [22]. Etoposide (VP-16), commonly used for treatment of non-Hodgkin lymphomas (NHL), induces intracellular oxidative stress by inhibiting topoisomerase II thereby generating DNA-strand breaks followed by cell apoptosis.

**Fig. 3:**
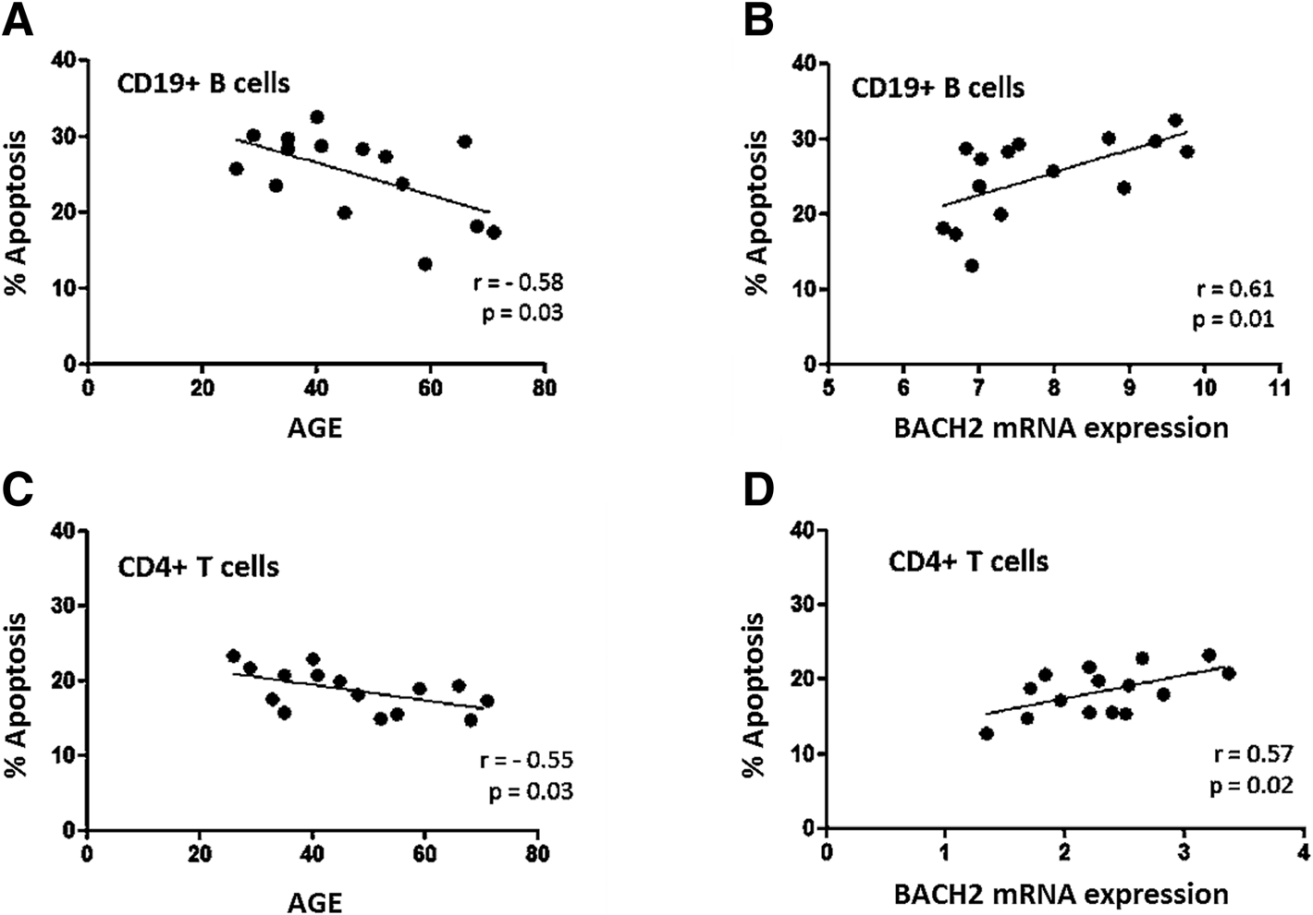
Age-related correlation of apoptosis (%) and BACH2 mRNA expression from healthy donors. Apoptosis decreases with age in both CD4+ T-cells and CD19+ B-cells (**a** and **c**). BACH2 downregulation correlated with apoptosis in function of age in CD19+ B cells and CD4+ T-cells (**b** and **d**). (Number of sample: CD4+: *n* = 15, CD19+: n = 15). The percentage of apoptosis was calculated using the difference in the percentage of cells (CD4+ or CD19+) expressing annexin-V after 24 h or 0 h of etoposide treatment

We examined etoposide-induced apoptosis in the CD4+ and CD19+ lymphocyte subpopulations. Apoptotic cells in these subpopulations were identified by immunostaining for CD45, CD19, CD3 and CD4 prior to annexin-V-FITC/IP. A reduction in apoptosis was observed with age in CD4+ T-cells and CD19+ B-cells (*p* = 0.03 in both subpopulations) (Fig. 3a, c and Additional file 6: Figure S1). As recently reported for BACH2-deficient mice [21], our data show that BACH2 downregulation is strongly correlated with a decrease in CD4+ T-cell and CD19+ B-cell apoptosis (*r* = 0.57 and 0.61) (Fig. 3b, d and Additional file 7: Figure S2).

### BACH2 and PRDM1 deregulation in T-cells and leukemic B-cells

We next examined *BACH2* and *PRDM1* gene expression in CD4+ and CD8+ T-cells and leukemic B-cells (CD19 + CD5+) from untreated CLL patients and age-matched HDs (> 50 years).

**Fig. 4:**
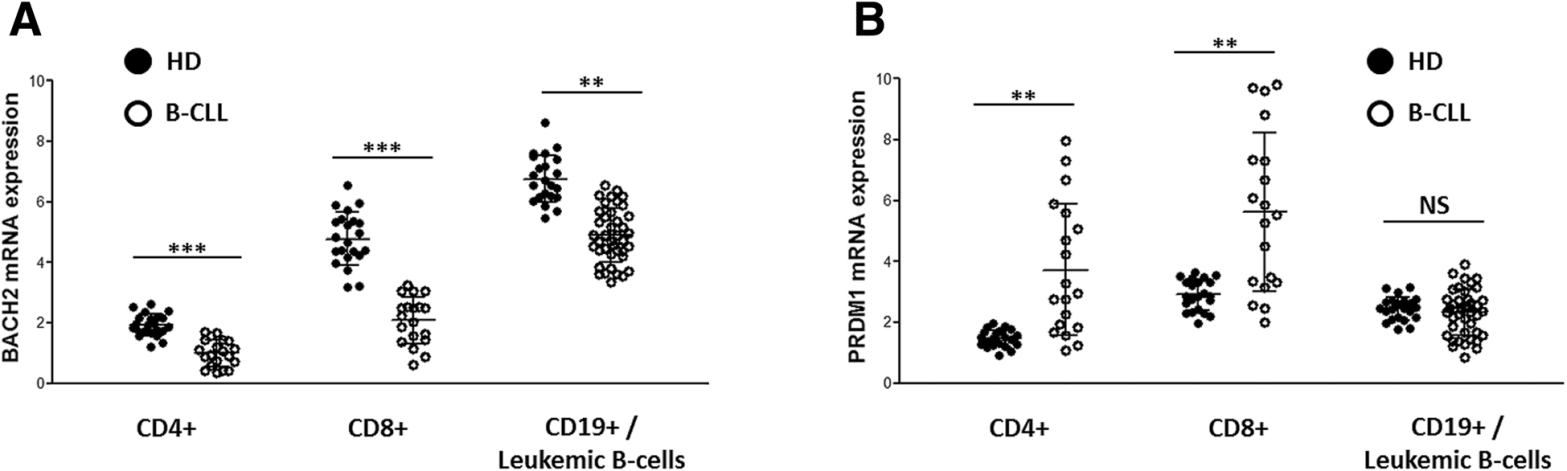
BACH2 and PRDM1 expression in T-cells and leukemic B-cells from chronic lymphocytic leukemia (CLL) patients and age-matched healthy donors (HD). **a** BACH2 mRNA expression in CD4+, CD8+ T-cells and leukemic B-cells from CLL patients versus healthy donors (*p* = 0.0006; 0.0003 and 0.0082, respectively). **b** PRDM1 mRNA expression in CD4+, CD8+ T-cells and leukemic B-cells from CLL patients versus healthy donors [*p* = 0.0034, 0.0012 and non-significant (NS), respectively]. HD: *n* = 22 for all subpopulations [median age: 67.8 years (51–86)], CLL patients [median age: 68.4 years (53–87)]: CD4+: *n* = 19; CD8+: *n* = 18; CD19: *n* = 41)

Decreases in *BACH2* mRNA expression were greater in CD4+ and CD8+ T-cells, as well as leukemic B-cell subpopulations from untreated CLL patient blood, compared with age-matched HDs (*p* = 0.0006; 0.0003 and 0.0083, respectively) (Fig. 4a). As expected, PRDM1 was significantly upregulated in the CD4+ T-cells and CD8+ T-cell subpopulations (*p* = 0.003 and 0.001, respectively) from CLL patients but not their leukemic B-cells (Fig. 4b).

**Fig. 5:**
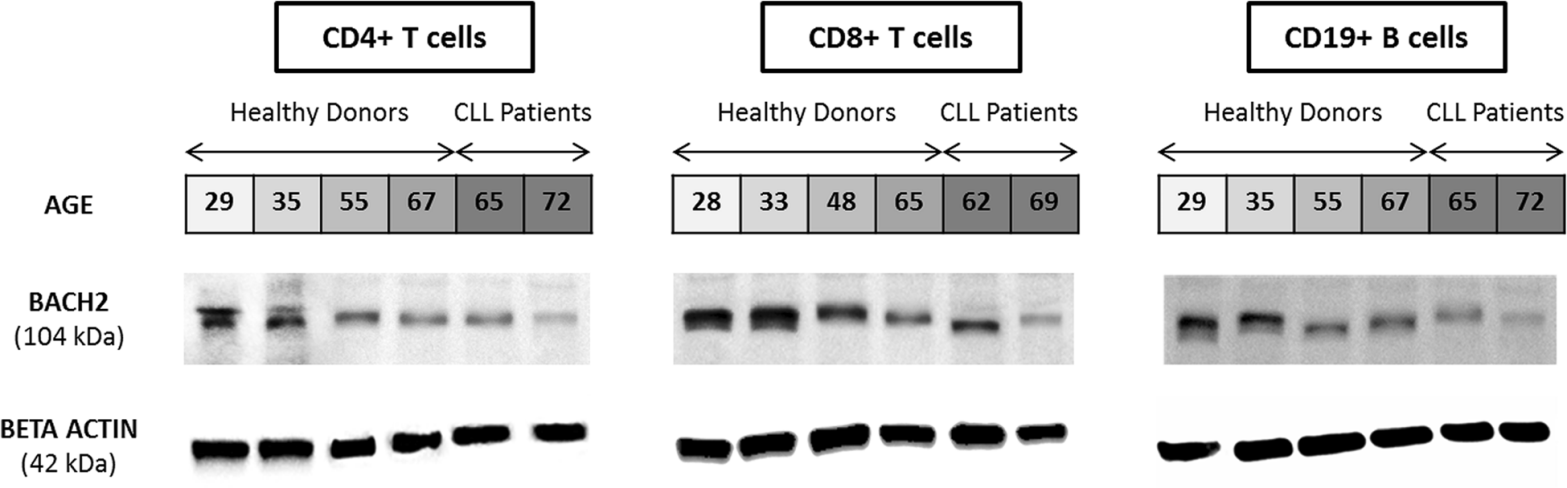
BACH2 protein expression in major lymphocyte subsets from healthy donors and CLL patients. Western blot analysis were performed using extracts from healthy donor and CLL patient purified CD4+, CD8+ and CD19+ cells (A, B and C, respectively)

Western blot analysis shows that BACH2 protein expression in CD4+ T-cells, CD8+ T-cells and CD19+ B-cells are significantly correlated with transcripts (Fig. 5). The lowest level of BACH2 mRNA expression in T-cells (both CD4+ and CD8+) and leukemic B-cells from CLL patients were confirmed at the protein level. The youngest HD had the highest BACH2 protein expression along with high expression levels of BACH2 mRNA. These observations confirm that age-related downregulation of *BACH2* gene expression is also translated to lower BACH2 protein expression.

## Discussion

Aging is known to affect the number of circulating lymphocytes and thereby impacts the adaptive immune response. The phenotypically different lymphocyte subpopulations have various immune functions which change by age. In this study, we focused on age-related changes in *BACH2* and *PRDM1* gene expression in the major lymphocyte subsets from HDs. Our aim was to understand how these two transcription factors are linked with decreasing immunity in the elderly.

BACH2 and PRDM1 clearly play important roles in immune homeostasis. BACH2 mediates immune homeostasis by suppressing PRDM1 in both CD4+ T-cells and B-cells [17, 29]. PRDM1 plays an important role in controlling the terminal differentiation in both effector B-cells and T-cells [18]. Our data show a dramatic downregulation of BACH2 expression (mRNA and protein) that parallels aging in the major lymphocyte subsets (helper and cytotoxic T-cells and B-cells). Expression levels of BACH2 are high in naïve and low in memory-effector CD4+ T-cells. As shown by previous studies, BACH2 decrease may release its suppression on PRDM1, which proportionally increases PRDM1 expression and thereby its activities that control terminal differentiation in effector B- and T-cells [17, 18, 29]. *BACH2* and *PRDM1* mRNA expression were inversely correlated in CD4+, CD4+ naïve, CD4+ memory T-cells, CD8+ T-cells and CD19+ B-cells, as expected. In the older HD group, up-regulation of PRDM1 mRNA expression was compatible with an increase in effector phenotypes. Although there were no significant changes in the number of naïve CD4+ T-cells, *BACH2* mRNA expression was significantly decreased in this subset, which likely affects some functions. In addition, we found an increase in *IL-4* gene expression (in parallel with PRDM1) correlated with age-related *BACH2* downregulation from CD4+ T-cells. This observation is compatible with the enhanced expression of innate/effector memory-related genes. BACH2 knockdown in CD4+ T-cells upregulated expression of Th2 genes including *IL-4*, *PRDM1* and *E4BP4* but not the Th1 gene *INFγ* [29]. Increased expression of effector cytokines such as IL-4 is known to be associated with inflammaging [10, 11]. This age-related loss of homeostasis is characterized by preferential differentiation of naïve CD4+ T-cells into Th2 and not due to *BACH2* downregulation. Furthermore, significant age-related changes were observed in the number and quality of CD8+ cytotoxic T-lymphocytes that known to play important roles in immunity, including viral clearance and elimination of aberrant cells [30, 31]. These findings suggested it is influencing age-related immune down-regulation.

A previous study done in CD4+ T-cell of mice found that BACH2 can induce apoptosis in response to oxidative stress by repressing the activation of Maf/AP-1 [21]. In similar results from our previous study, we demonstrated that BACH2 could play a role as a tumor suppressor gene by increasing the apoptosis of CD4+ T-cells in vitro. In the same study, silencing of BACH2 in Jurkat CD4+ T-cells clones treated with etoposide markedly reduced apoptosis compared with healthy control [32]. Moreover, several reports pinpointed BACH2 as a candidate tumor suppressor gene in the B cell compartment by showing frequent loss of heterozygosity in non-Hodgkin lymphomas and a reduction of clonogenicity with increased sensitivity to apoptosis in BACH2 over-expressing Raji cells [33]. Etoposide-induced oxidative stress experiments revealed an age-related correlation between BACH2 down-regulation and resistance to apoptosis in the major lymphocyte subsets. In our healthy cohort, this decline in apoptosis was apparent in two major lymphocyte subsets (CD4+ T-cells and B-cells) and strongly correlated with decreased BACH2 expression. Our data suggest that age-related decreases in *BACH2* gene expression could reduce apoptosis in response to oxidative stress. On the other hand, PRDM1 can induce cell cycle arrest but not apoptosis [34]. Although high *PRDM1* mRNA expression levels were detected in the older HD group, it was not directly involved in apoptosis.

Following the remarkable results on BACH2 and PRDM1 that could be associated with age-related immune deterioration in HDs, we analyzed *BACH2* and *PRDM1* gene expression in untreated CLL patients who were not on any medication that might influence their immune response. Interestingly, in leukemic B-cells from our CLL patients, BACH2 decreases while no changes in PRDM1 expression were detected in comparison with age-matched HDs. BACH2 deficiency could cause deregulated checkpoint control signaling and lead to B-cell precursor leukemia cells [35]. Furthermore, the malignant B-cell is associated with failure of inducing PRDM1, a critical regulator of differentiation into plasma cells. Epigenetic analyses show both DNA methylation and histone modifications associated with PRDM1 transcriptional control elements [24]. BACH2 interact with histone deacetylase 3-containing co-repressor complexes (NCoR1, NCoR2, Tbl1x and Rif1) in B-cells to regulate the stage-specific expression of PRDM1 by writing epigenetic modifications at the PRDM1 locus [36]. Moreover, approximately 23–50% of lymphoid malignancies have *PRDM1* gene alterations [37]. In DLBCL, there is a lack of BLIMP1 (PRDM1) protein expression even though *PRDM1* mRNA is produced, which has been linked with incomplete transcription [38]. These findings could explain why no significant change of *PRDM1* gene expression was observed in leukemic B-cells compared to age-matched HDs.

Decreasing BACH2 levels in T-cells and leukemic B-cells reduces their ability to undergo apoptosis. A BACH2 deficiency has also been shown to induce an increase in IL-4-producing CD4+ T-cells [29]. This was demonstrated by our previous work showing that IL-4 production is enhanced in CD3-CD4+ T-cells harboring a 6q deletion with low *BACH2* mRNA expression [39, 40]. This increased PRDM1 and IL-4 has been shown to protect leukemic cells from death by apoptotic cell death [41]. Our observation also suggests that BACH2 downregulation in T-cells and B-cells from CLL patients may favor their resistance to apoptosis.

## Conclusion

Our data show that downregulation of BACH2 and upregulation of PRDM1 in human immune cells are significantly correlated with aging and may play a role in immunosenescence. We further demonstrate that BACH2 downregulation in normal lymphocytes increases age-related resistance to apoptosis. These alterations were even more pronounced in T- and B-cells from CLL patients. Both observations suggest the potential role for BACH2 and PRDM1 in immunosenescence process.

## Additional files

Additional file 1: **Table S1.** Details of PCR primers used for RT-qPCR. (XLSX 9 kb)

Additional file 2: **Table S2.** Antibodies used in the Flow cytometry analysis. (XLSX 9 kb)

Additional file 3: **Table S3.** Regression analysis of major lymphocyte subset modifications from healthy donors versus age (*n* = 60). (XLSX 9 kb)

Additional file 4: **Table S4.** Regression analysis of *BACH2* mRNA expression in major lymphocyte subsets versus age. (XLSX 9 kb)

Additional file 5: **Table S5.** Regression analysis of *PRDM1* mRNA expression in major lymphocyte subsets versus age. (XLSX 9 kb)

Additional file 6: **Figure S1.** Gene expression of BACH2 and effector memory-related genes: PRDM1, IL-4 and IFNγ in CD4+ T-cells from healthy donors. (A) BACH2 mRNA expression decreased with age in CD4+ (*r* = − 0.67, *p* < 0.0001) (B) PRDM1 mRNA expression increased with age in CD4+ (*r* = − 0.61, *p* = 0.0001), (C) IL-4 mRNA expression increased with age in CD4+ (*r* = − 0.66, *p* < 0.0001), (D) IFNγ mRNA expression showed non-significant change with age in CD4+ (*r* = − 0.28, *p* = 0.0641). Significant *p* values (< 0.05) and Spearman r values are shown. (TIF 6567 kb)

Additional file 7: **Figure S2.** Apoptosis in function of age. PBMCs from HDs were incubated with 50 μM etoposide for 24 h. Apoptotic cellular subpopulations were identified by immunostaining for CD45, CD19, CD3 and CD4 prior to annexin-V-FITC/IP. (TIF 4876 kb)

## Abbreviations

BACH2: BTB and CNC homology 1, basic leucine zipper transcription factor 2
CLL: Chronic Lymphocytic Leukemia
HD: Healthy donor
NK: natural killer
PBMC: peripheral blood mononuclear cells
PRDM1: PR domain zinc finger protein 1
TNF: Tumor necrosis factor
