# Supplementary figures and images for: Age-related changes in the BACH2 and PRDM1 genes in lymphocytes from healthy donors and chronic lymphocytic leukemia patients
Source: BMC Cancer. 2019 Jan 17;19:81. doi: 10.1186/s12885-019-5276-2 (PMC6337793; doi:10.1186/s12885-019-5276-2)

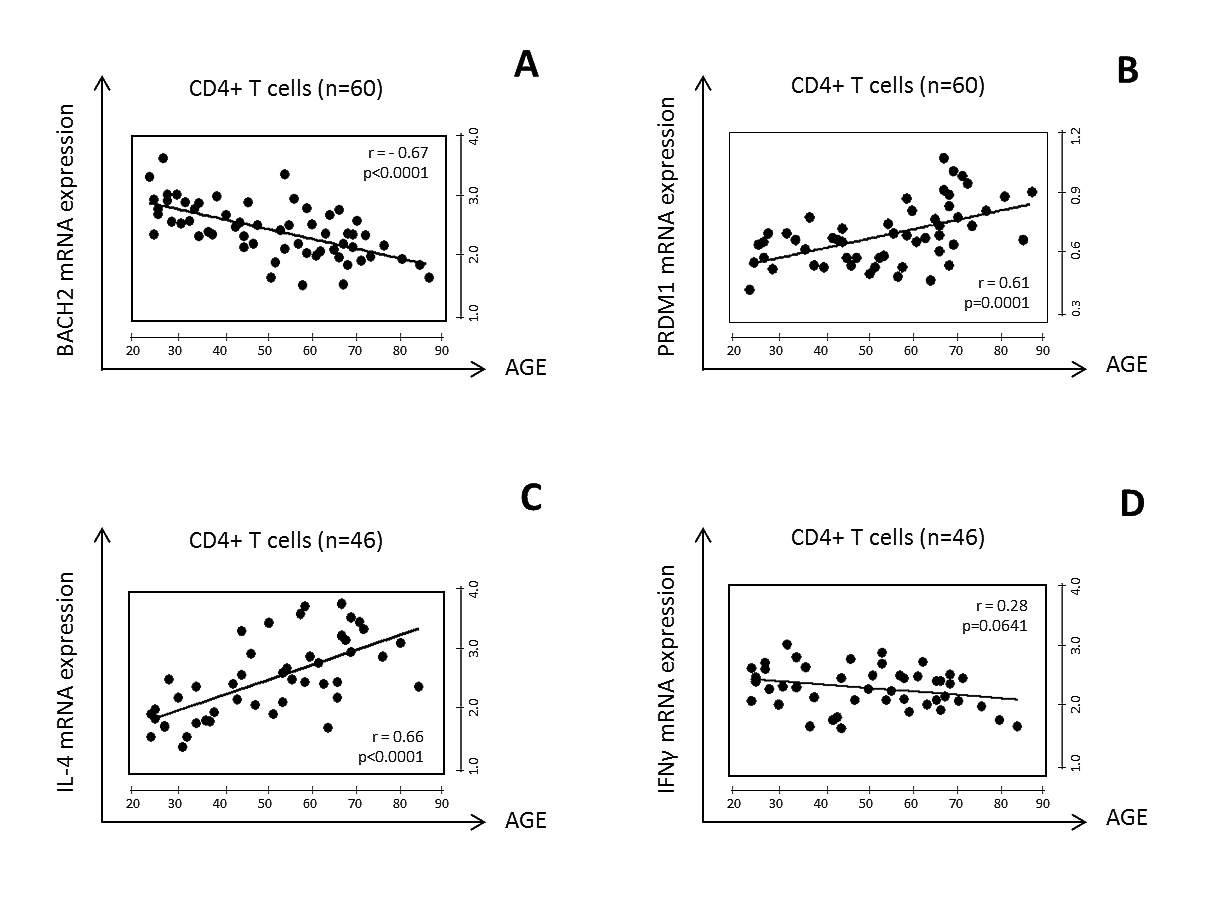

Supplement: Supplementary file 6 — Figure S1. Gene expression of BACH2 and effector memory-related genes: PRDM1, IL-4 and IFNγ in CD4+ T-cells from healthy donors. (A) BACH2 mRNA expression decreased with age in CD4+ (r = − 0.67, p < 0.0001) (B) PRDM1 mRNA expression increased with age in CD4+ (r = − 0.61, p = 0.0001), (C) IL-4 mRNA expression increased with age in CD4+ (r = − 0.66, p < 0.0001), (D) IFNγ mRNA expression showed non-significant change with age in CD4+ (r = − 0.28, p = 0.0641). Significant p values (< 0.05) and Spearman r values are shown. (TIF 6567 kb) [file 12885_2019_5276_MOESM6_ESM.tif]

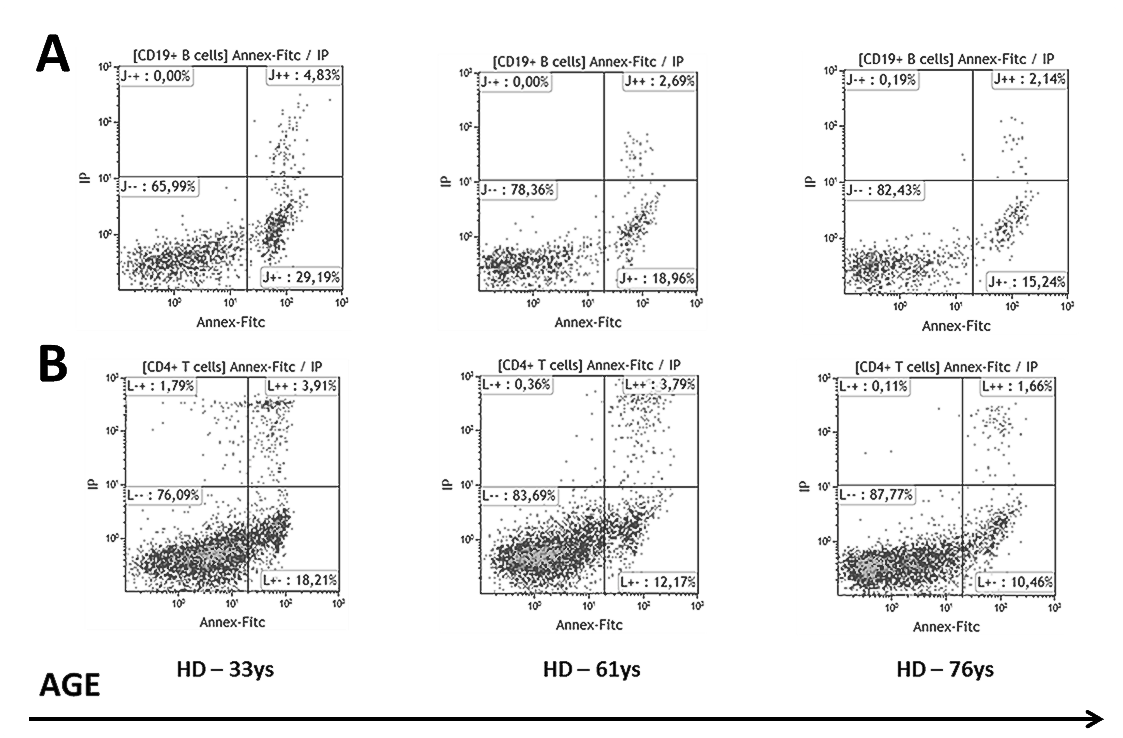

Supplement: Supplementary file 7 — Figure S2. Apoptosis in function of age. PBMCs from HDs were incubated with 50 μM etoposide for 24 h. Apoptotic cellular subpopulations were identified by immunostaining for CD45, CD19, CD3 and CD4 prior to annexin-V-FITC/IP. (TIF 4876 kb) [file 12885_2019_5276_MOESM7_ESM.tif]
